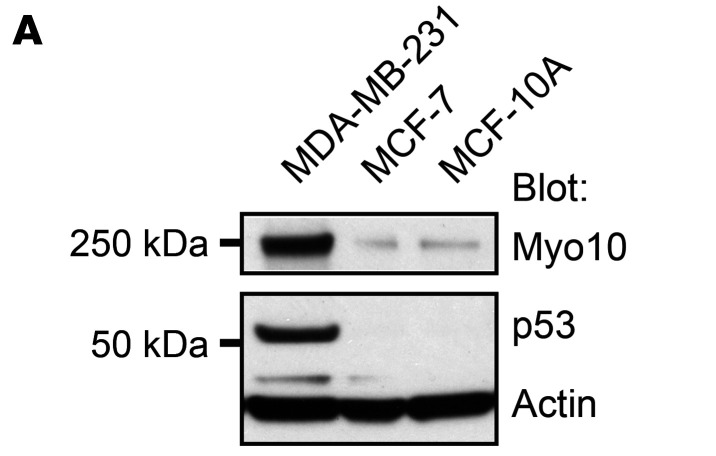# Corrigendum to Mutant p53–associated myosin-X upregulation promotes breast cancer invasion and metastasis

**DOI:** 10.1172/JCI201379

**Published:** 2025-12-01

**Authors:** Antti Arjonen, Riina Kaukonen, Elina Mattila, Pegah Rouhi, Gunilla Högnäs, Harri Sihto, Bryan W. Miller, Jennifer P. Morton, Elmar Bucher, Pekka Taimen, Reetta Virtakoivu, Yihai Cao, Owen J. Sansom, Heikki Joensuu, Johanna Ivaska

Original citation: *J Clin Invest*. 2014;124(3):1069–1082. https://doi.org/10.1172/JCI67280

Citation for this corrigendum: *J Clin Invest*. 2025;135(23):e201379. https://doi.org/10.1172/JCI201379

The authors recently became aware that in Figure 6A, the Myo10 blot was incorrect and was an inadvertent duplication of the p53 blot in Figure 6A. The correct figure panel, based on the original source data, is provided below.

The authors regret the error.

## Figures and Tables

**Figure 6A:**